# Study of flexural strength of concrete containing mineral admixtures based on machine learning

**DOI:** 10.1038/s41598-023-45522-4

**Published:** 2023-10-23

**Authors:** Yue Li, Yunze Liu, Hui Lin, Caiyun Jin

**Affiliations:** 1 Key Laboratory of Urban Security and Disaster Engineering of Ministry of Education, Beijing Key Laboratory of Earthquake Engineering and Structural Retrofit, Beijing University of Technology, No. 100 Pingleyuan, Chaoyang District, Beijing, 100124 China; 2 Faculty of Science, Beijing University of Technology, No. 100 Pingleyuan, Chaoyang District, Beijing, 100124 China

**Keywords:** Civil engineering, Structural materials

## Abstract

In this paper, the prediction of flexural strength was investigated using machine learning methods for concrete containing supplementary cementitious materials such as silica fume. First, based on a database of suitable characteristic parameters, the flexural strength prediction was carried out using linear (LR) model, random forest (RF) model, and extreme gradient boosting (XGB) model. Subsequently, the influence of each input parameter on the flexural strength was analyzed using the SHAP model based on the optimal prediction model. The results showed that LR, RF, and XGB enhanced the accuracy of forecasting sequentially. Among the characteristic parameters, the most significant effect on the flexural strength of concrete is the water-binder ratio, and the water-binder ratio shows a negative correlation with flexural strength. The effect of maintenance age on flexural strength is second only to the water-binder ratio, and it shows a positive trend. When the amount of fly ash is less than 40% and the amount of slag or silica fume is less than 30%, the correlation between the amount of supplementary cementitious materials and flexural strength fluctuates and a positive peak in flexural strength is observed. However, at a dosage greater than the above, the supplementary cementitious materials all reduce flexural strength. The interaction interval and the degree of interaction between the supplementary cementitious materials and the cement content also differ in predicting flexural strength.

## Introduction

The mechanical properties of concrete have been of great interest^1,2^. Among the many mechanical properties, strength is one of the most basic indicators. The study of concrete strength can be traced back to the beginning of the invention of concrete materials. Usually, the strength of concrete includes compressive strength and flexural strength, etc.^3,4^. For some structural members with special force and support requirements, the flexural strength of concrete must meet the requirements, the in-depth study of which is extremely important^5,6^.

During the study of concrete flexural strength, specimens were usually tested on the basis of changing the raw material ratio, curing conditions, and the curing age^5,7,8^. These studies have greatly enriched the understanding of the factors influencing the flexural strength. Among the many factors affecting the flexural strength of concrete, the composition of raw materials is one of the most basic and important factors^9,10^. Among the raw materials, the connection between cement and strength is more complex and important compared to other raw materials such as aggregates because the hydration products of cement are directly related to strength^11,12^. However, the massive use of cement will produce more greenhouse gases such as carbon dioxide, which aggravates environmental pollution and contradicts the current promotion of energy saving and carbon neutrality^13^. This has led to a gradual shift of attention to the development of cementitious materials with a certain cementing capacity and low carbon emissions. Compared to cement, supplementary cementitious materials such as fly ash, slag, silica fume, and metakaolin are more environmentally friendly both in preparation and use^14–17^. This makes it important to study in depth the effect of supplementary cementitious materials on the mechanical properties (e.g., strength) of concrete. Currently, there have been many studies on the strength of concrete containing supplementary cementitious materials, as summarized in Table 1.

**Table 1:** Summary of the study on the strength of concrete containing supplementary cementitious materials.

No	SCM type	Strength properties	Researchers	Comments
1	Slag	Compressive and flexural strength	Chore	^18^
2	Fly ash	Compressive and flexural strength	Siddique	^19^
3	Silica sume	Flexural strength	Xie	^20^
4	Slica fume	Flexural strength	Sabir	^21^
5	Fly ash	Compressive strength	Kim	^22^
6	Slag	Compressive and flexural strength	Lee	^23^
7	Slag	Flexural strength	Tripathi	^24^
8	Fly ash	Compressive and flexural strength	Padavala	^25^
9	Slica fume	Flexural strength	Bhanja	^26^
10	Fly ash	Flexural strength	Cao	^27^
11	Rice husk	Compressive and flexural strength	Sathawane	^28^
12	Metakaolin	Compressive and flexural strength	Abdelmelek	^29^

The above studies have provided a significant boost to the related field and have led to a better understanding of it. However, most of these studies have been carried out by traditional experimental means, i.e., by controlling specific ages, specific water-binder ratios, etc., and obtaining specific values using a press machine. This will make the concrete containing supplementary cementitious materials in some projects with large volume and very high strength characteristics requirements, which will increase the experimental labor and material as well as time costs. Moreover, due to the influence of multiple factors, the final test results may be different from the actual engineering application. Therefore, the search for a concrete strength research method that can integrate multiple strength influencing factors with high efficiency and accuracy is on the agenda. With the development of computer science, some researchers have introduced machine learning into the study of concrete strength^30–34^. For example, Nguyen et al.^35^ studied the compressive strength of concrete by machine learning model. Zeng et al.^36^ used deep learning theory to predict achieved accurate prediction of compressive strength of concrete. However, most of the aforementioned studies have focused on concrete compressive strength. Few studies have been conducted on the flexural strength of concrete using machine learning, and no studies have been reported on the flexural strength of concrete containing supplementary cementitious materials. Therefore, there is a need to conduct scientific machine learning modeling studies for the flexural strength of concrete containing supplementary cementitious materials to promote the development of flexural strength of low-carbon concrete.

In view of the above, this study selected fly ash, slag, and silica fume, which are three widely used supplementary cementing materials at present^37–40^. The existing studies were synthesized and a database covering the appropriate feature parameters was established. Based on this database, modeling studies were conducted using the linear (LR) model, random forest (RF) model, and extreme gradient boosting (XGB) model. The purpose of the above machine learning model selection is first to verify whether there is a linear relationship between various feature parameters, and then select two integrated machine learning models that are currently widely used for in-depth analysis. On the basis of the optimization model, the characteristic features were investigated based on the Shapley additive interpretation (SHAP).

## Database analysis

### Characteristic parameter establishment

There are numerous elements affecting the flexural strength of concrete (F_S), including water-binder ratio, aggregate content, etc. For concrete containing supplementary cementitious materials such as fly ash, slag, and silica fume, the admixture of supplementary cementitious materials is also an important influencing factor. Therefore, this study summarizes the factors influencing the strength of concrete with supplementary cementitious materials according to the possible differences in the process of making and maintaining concrete. First, in the preparation stage of concrete with supplementary cementitious materials, cement as the main cementitious material, the influence of cement content (cem_con) on the flexural strength of concrete cannot be ignored. Taking the cement admixture as a benchmark, the admixture of fly ash (FA_con), slag (S_con), and silica fume (SF_con) as alternative cement materials in the concrete is also the focus of this study. As an important participant in the hydration process of cementitious materials that cannot be ignored, the water content has an important effect on the strength. Therefore, the water-binder ratio (w_b) is also a characteristic focused on the study. Aggregate, as the component with the largest proportion of the three-phase composition of concrete, also has an important influence on the flexural strength of concrete, so fine aggregate content (Fine_A_con) and coarse aggregate content (Coarse_A_con) are set as characteristic parameters as well. During the curing of concrete, the hydration process of the cementitious material changes with the increase of curing time, so the curing age (Age) also has an important influence on the flexural strength. It is worth noting that the curing temperature and the curing humidity also affect the hydration of the cementitious materials during the curing of concrete specimens, which in turn affects the flexural strength of concrete, but these two are not considered characteristic parameters in this study. The curing temperatures of the selected concrete specimens were in the range of 20 ± 2 °C and the relative humidity fluctuated in the range of 60 ± 5%.

### Determination of the database

When using machine learning methods for predictive modeling of material properties, a database covering all feature parameters with a sufficient amount of data is required. A good database contributes to the successful implementation of machine learning modeling and helps to improve the accuracy of subsequent feature parameter analysis. In this study, eight sets of input parameters (w_b, cem_con, SF_con, S_con, FA_con, Fine_A_con, Coarse_A_con, Age) and one set of output parameters (F_S) were set according to the influencing factors given in "Characteristic parameter establishment" section. Thirty-one papers related to the flexural strength of concrete containing supplementary cementitious materials were read and summarized^18–21,23–29,41–60^, and 373 valid data sets were compiled. The data on flexural strength of 373 groups of concrete were counted, and the mathematical characteristics of the input and output parameters were obtained as shown in Table 2. It can be seen that the data distribution interval corresponding to each characteristic parameter is relatively wide, and the amount of data covered is sufficient to avoid the adverse effects of data concentration on the model prediction results to a certain extent.

**Table 2:** Statistical results of parameter characteristics.

	No	Parameters	Unit	Minimal	Maximum	Average	Q-25%	Q-75%	Std
Input	1	w_b		0.26	0.6	0.38	0.33	0.45	0.08
	2	cem_con	kg/m^3^	110	591	330	240	420	108
	3	SF_con	%	0	53.1	12.94	0	16.03	8.91
	4	S_con	%	0	57.8	22.12	0	26.8	14.57
	5	FA_con	%	0	65.4	24.28	0	31.2	17.48
	6	Fine_A_con	kg/m^3^	200	970	665	616	747	132.09
	7	Coarse_A_con	kg/m^3^	400	1320	1090	978	1200	163.79
	8	Age	d	3	365	38	7	28	57.3
Output	1	F_S	MPa	0.2	16.8	6.15	4.1	7.6	2.65

The requirements for the database are to ensure that, in addition to the quantity and quality of the dataset, there are no significant linear relationships between the feature parameters. When the linear relationship between the feature parameters is strong, it will affect the model prediction accuracy. In order to characterize the linear relationship between different feature parameters, statistical analysis is performed using Pearson correlation coefficient (PCC) in Eq. (1). The *i* is data sample; *m* is the number of *i*; *y*_*i*_, ${y}_{i}{\prime}$ are the true and predicted values; ${\overline{y} }_{i}$, ${\overline{y} }_{i}{\prime}$ are the average values of the above. Usually, the correlation between the two data sets can be considered negligible for the exactness of the model predicted outcome when the PCC value is between -0.4 and 0.4.

1 $$P{\text{CC}} = {{\sum\nolimits_{i = 1}^{m} {\left( {y_{i} - \overline{y}} \right)\left( {y^{\prime}_{i} - \overline{y^{\prime}}} \right)} } \mathord{\left/ {\vphantom {{\sum\nolimits_{i = 1}^{m} {\left( {y_{i} - \overline{y}} \right)\left( {y^{\prime}_{i} - \overline{y^{\prime}}} \right)} } {\sqrt {\sum\nolimits_{i = 1}^{m} {(y_{i} - \overline{y})^{2} } } \sqrt {\sum\nolimits_{i = 1}^{m} {(y^{\prime}_{i} - \overline{y}^{\prime})^{2} } } }}} \right. \kern-0pt} {\sqrt {\sum\nolimits_{i = 1}^{m} {(y_{i} - \overline{y})^{2} } } \sqrt {\sum\nolimits_{i = 1}^{m} {(y^{\prime}_{i} - \overline{y}^{\prime})^{2} } } }}$$

**Figure 1:**
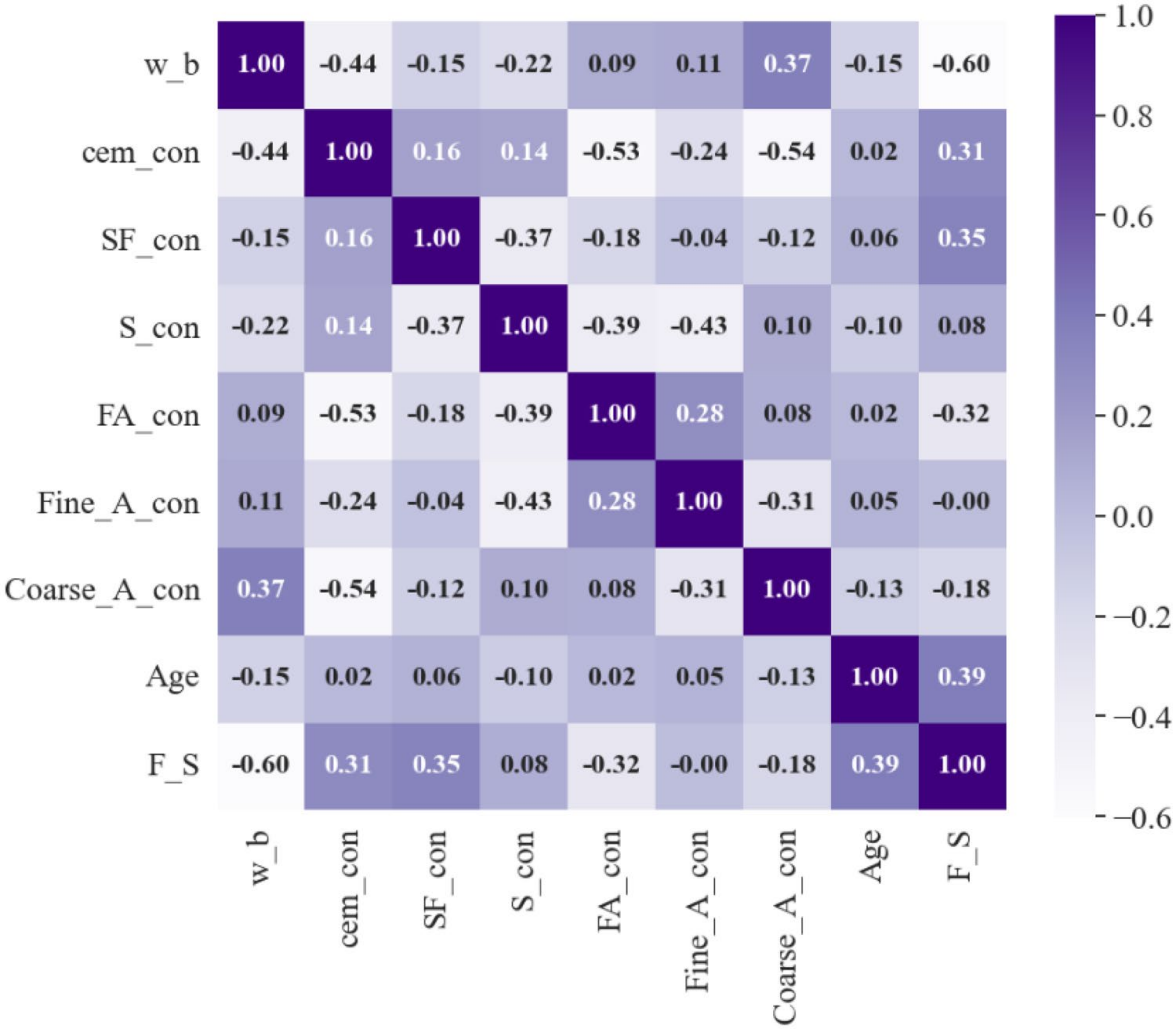
Relationship diagram of each parameter.

The data hot map of PCC values for all input parameters and output parameters is shown in Fig. 1. From the hot map, it can be seen that the vast majority of the input parameters exhibit weak correlation with each other, except for individual input parameters whose PCC values are not between -0.4 and 0.4. This indicates that the data in the database used in this study are superior. Comparing the correlations between the input and output parameters, it was found that only the water-binder ratio had a significant correlation with the flexural strength of concrete, while the remaining seven groups of input parameters showed a low correlation with the flexural strength of concrete.

## Machine learning

### Machine learning models

#### LR model

**Figure 2:**
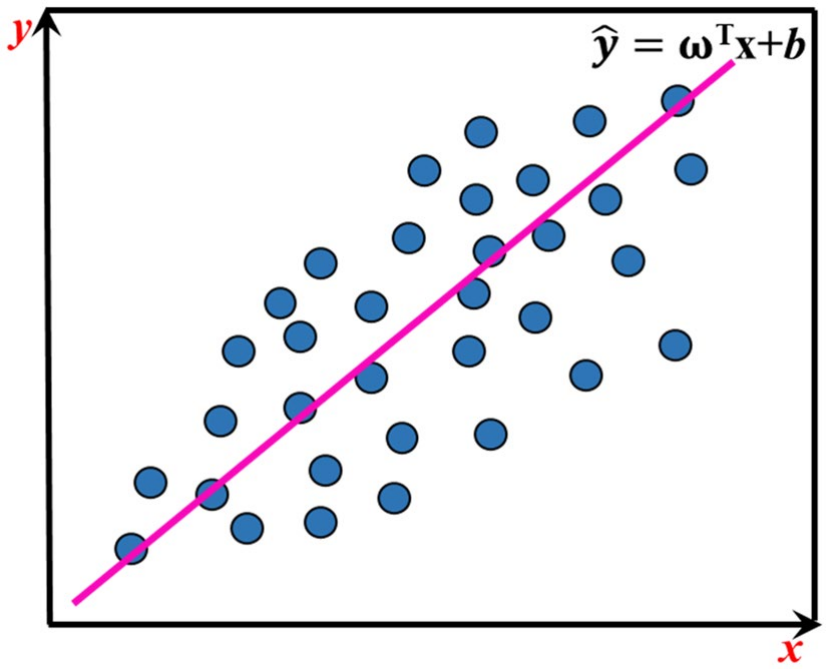
LR model principle.

Many machine learning models have been successfully validated in the study of performance prediction of concrete materials. The most basic model is the LR model, which has a simple modeling process, simple analytical ideas, and fast computation speed, and was widely used before the integrated machine learning system was proposed. The calculation principle of the LR model is shown in Fig. 2.

#### RF model

Integrated algorithmic models, as emerging machine learning models, possess more sophisticated computational procedures that can significantly improve prediction accuracy compared to linear model systems. Machine learning is accomplished by constructing multiple learners that adopt specific strategies. The purpose of the integrated algorithm is to construct a strong learning machine from multiple weak learners. This leads to a better analysis of various parameter features^17^.

The base learner chosen for modeling the flexural strength of concrete through the RF model is the decision tree. Autonomous aggregation algorithm (bootstrap aggregation) for learners^61^. When using the bagging method for calculations. During processing, strength learners are obtained by processing many weaker learners. Subsequently, the average value is processed.

**Figure 3:**
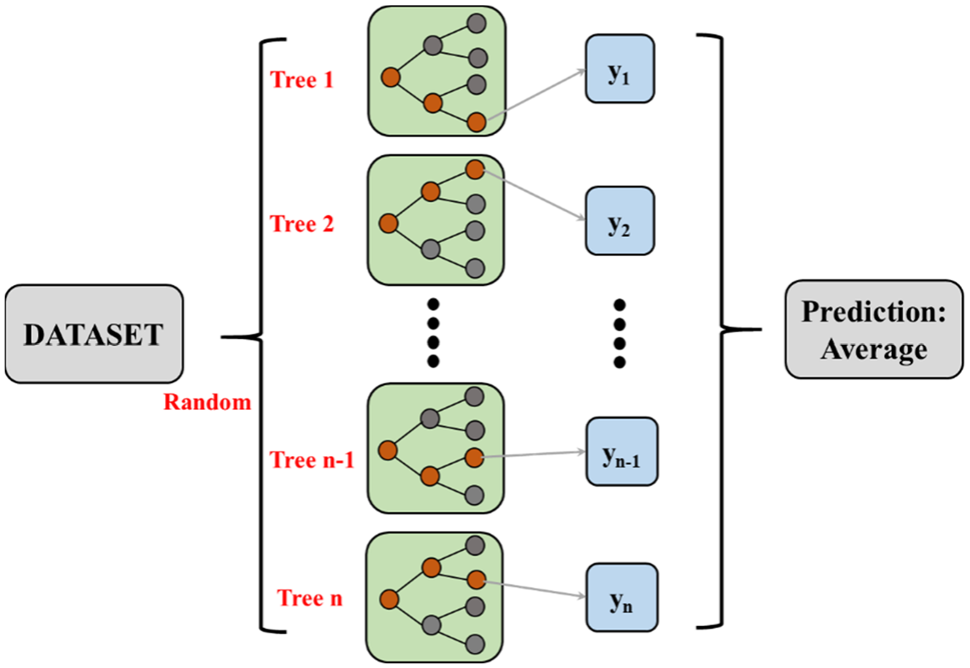
RF model principle.

This model is an extended extension of the bag method in which the schematic diagram is presented in Fig. 3. As shown in the figure, the database of the RF model is divided into multiple branches. After considering the feature performance of each branch, the results of the altered model are obtained by averaging^61^.

#### XGB model

**Figure 4:**
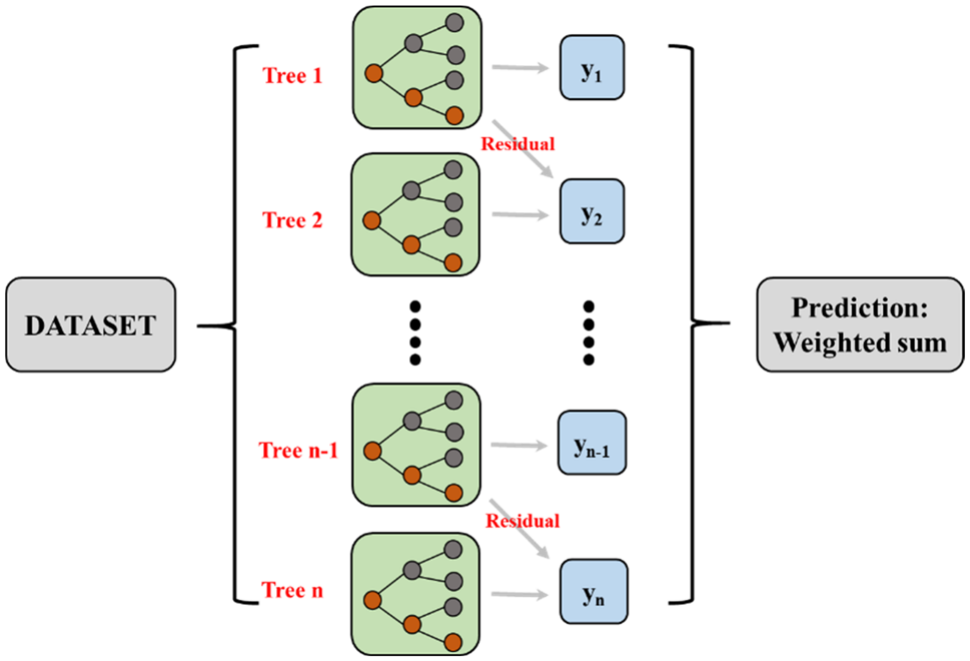
XGB model principle.

Among centralized machine learning systems, the XGB model is a rapidly developing and widely used efficient learning model because it's fast, flexible and lightweight. The XGB model was also utilized as a decision tree to serve as the basic collector for machine learning. The integrated learning method used in the XGB model is the Boosting^35^. Figure 4 shows the schematic diagram of the method.

In the computational processing using the XGB model, the output parameters are obtained from a base learner consisting of *V* number of tree structures (*f*_*1*_, *f*_*2*_, …, *f*_*V*_), as shown in Eq. (2).

2 $$y_{i} = \sum\limits_{v = 1}^{V} {f_{V} \left( {x_{i} } \right)}$$

Subsequently, in order to obtain the objective function of the above function at the minimization iteration *u*, it is necessary to calculate the minimum loss function as shown in Eq. (3) by:

3 $$\chi^{a} = \sum\limits_{i = 1}^{m} {H\left( {y_{i} ,Y_{i}^{(u - 1)} } \right)} + f_{u} (X_{i} )) + \phi (f_{u} )$$

where $\varnothing$ denotes the regularization. To obtain the minimized objective function at *u* (Eq. (4)), it is necessary to use the Taylor approximation.

4 $$\overline{{\chi^{a} }} = \sum\limits_{i = 1}^{m} {\left[ {L_{i} f_{u} (X_{i} ) + {{P_{i} f_{u}^{2} (X_{i} )} \mathord{\left/ {\vphantom {{P_{i} f_{u}^{2} (X_{i} )} 2}} \right. \kern-0pt} 2}} \right]} + \phi (f_{u} )$$

The expressions for *L*_*i*_ and *P*_*i*_ in the above equation are shown in Eq. (5) and Eq. (6), respectively.

5 $$L_{i} = \partial_{{Y_{i}^{(u - 1)} }} H(y_{i} ,Y_{i}^{(u - 1)} )$$

6 $$P_{i} = \partial_{{_{{Y_{i}^{(u - 1)} }} }}^{2} H(y_{i} ,Y_{i}^{(u - 1)} )$$

In the XGB model, when the number of leaves of *f*_*u*_ is assumed to be *V* and *r*_*w*_ denotes the weight of the *w*th leaf, the regularization formula has the following expression:

7 $$\phi (f_{u} ) = \lambda V + {{\gamma \sum\limits_{w = 1}^{V} {r_{w}^{2} } } \mathord{\left/ {\vphantom {{\gamma \sum\limits_{w = 1}^{V} {r_{w}^{2} } } 2}} \right. \kern-0pt} 2}$$

where *λ* and *γ* are constants. Finally, when the form of the tree structure is fixed, e.g., the optimal prediction corresponding to the tree structure d can be obtained by Eq. (8).

8 $$\overline{{\chi^{a} }} (d) = {{ - \left[ {(\sum\limits_{w = 1}^{V} {{{(\sum\limits_{{i \in T_{w} }} {L_{i} } )^{2} } \mathord{\left/ {\vphantom {{(\sum\limits_{{i \in T_{w} }} {L_{i} } )^{2} } {\sum\limits_{{i \in T_{w} }} {P_{i} + \gamma ) + \lambda V} }}} \right. \kern-0pt} {\sum\limits_{{i \in T_{w} }} {P_{i} + \gamma ) + \lambda V} }}} } \right]} \mathord{\left/ {\vphantom {{ - \left[ {(\sum\limits_{w = 1}^{V} {{{(\sum\limits_{{i \in T_{w} }} {L_{i} } )^{2} } \mathord{\left/ {\vphantom {{(\sum\limits_{{i \in T_{w} }} {L_{i} } )^{2} } {\sum\limits_{{i \in T_{w} }} {P_{i} + \gamma ) + \lambda V} }}} \right. \kern-0pt} {\sum\limits_{{i \in T_{w} }} {P_{i} + \gamma ) + \lambda V} }}} } \right]} 2}} \right. \kern-0pt} 2}$$

Equation (8), also known as the quality evaluation function, can be used to evaluate the optimal weight results of the base learner with a number of leaves. More explanation of the XGB model can be found in the introduction given in the literature^62^.

### Evaluation indicators

Different machine learning models have different computational procedures, which leads to differences in the prediction results when using different models to predict the same properties. In order to scientifically judge the prediction effectiveness of the models in predicting the flexural strength, five metrics commonly used in mathematical statistics were used. The first is the coefficient of determination, the formula for R^2^ given in Eq. (9). In mathematical statistics, R^2^ is one of the most commonly judged indicators. By normalizing the residuals of the data, a coefficient of determination value with a size between 0 and 1 can be generated, and the closer the value of the coefficient of determination is to 1, the better the model's prediction is indicated. Equation (10) denotes the mean absolute error (MAE). Equation (11) and Eq. (12) denote the mean squared error (MSE) and root mean squared error (RMSE), both of which can be used to judge the excellence of the model by evaluating the magnitude of the change in data development during the prediction process. Also, PCC is an important criterion for analyzing linear relationships. The smaller the calculated values for MAE, MSE and RMSE, the more reliable the model is. The closer the value of PCC is to 1, the more reliable the model is.

9 $$R^{2} = 1 - \frac{{\sum\nolimits_{x = 1}^{n} {(y_{x} - y^{\prime}_{x} )^{2} } }}{{\sum\nolimits_{x = 1}^{n} {(y_{x} - \overline{y})^{2} } }}$$

10 $$MAE = \frac{1}{n}\sum\nolimits_{x = 1}^{n} {\left| {y_{x} - y^{\prime}_{x} } \right|}$$

11 $$MSE = \frac{1}{n}\sum\nolimits_{x = 1}^{n} {(y_{x} - y^{\prime}_{x} )^{2} }$$

12 $$RMSE = \sqrt {\frac{1}{n}\sum\nolimits_{x = 1}^{n} {(y_{x} - y^{\prime}_{x} )^{2} } }$$

### Flow chart

In the prediction of flexural strength, this study started with a comprehensive analysis of the factors influencing flexural strength. In addition to the non-essential factors and common conditions such as temperature and humidity, eight input parameters including w_b and cem_con, and the output parameter F_S were identified. Subsequently, the available test results of flexural strength of concrete containing supplementary cementitious materials were statistically analyzed and a database containing 373 sets of data was established. Subsequently, the linear relationship between the characteristic parameters was analyzed based on the PCC calculation results to ensure the reliability of the database established in this study.

When using machine learning models for flexural strength prediction to determine the most suitable machine learning model. In this study, three models were selected. Out of the total data, seventy percent is training data and thirty percent is test data. After parameter optimization of each model, five mathematical and statistical evaluation metrics such as R^2^ and MAE were used to evaluate the prediction effectiveness of various models with respect to the model calculation results.

**Figure 5:**
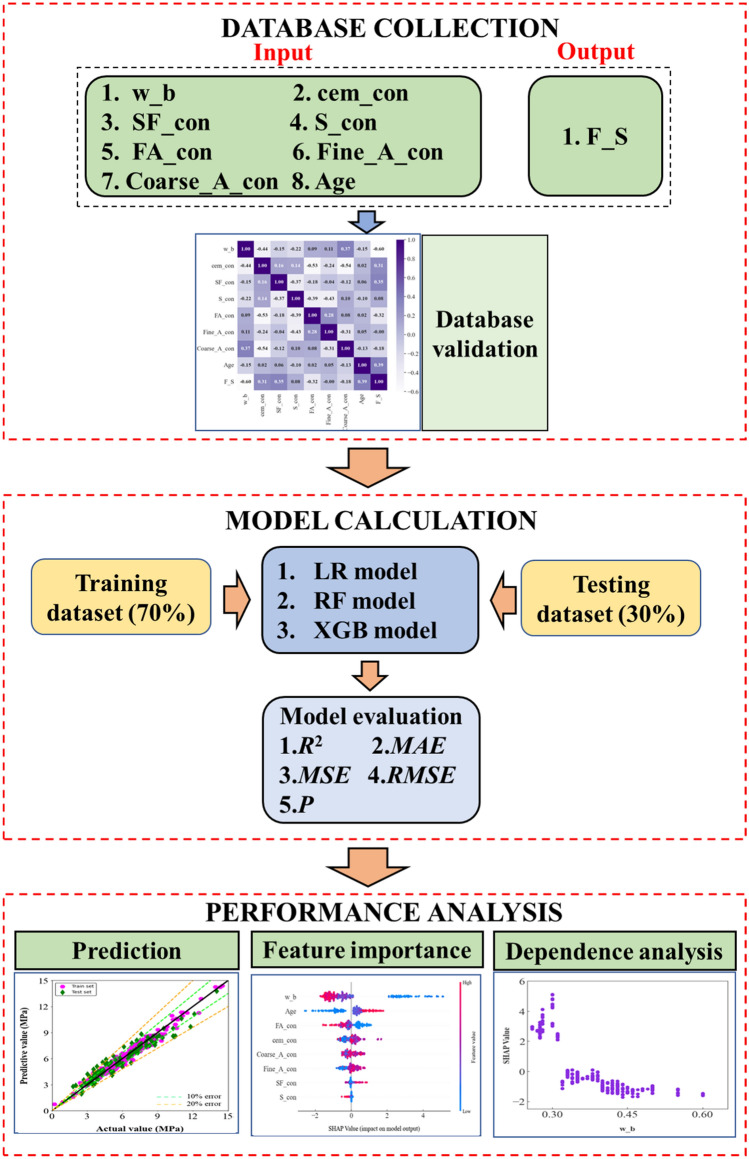
Sketch of the whole process of analyzing and processing in this study.

Finally, in order to determine the effect of each feature parameter, this study illustrates the correlation of the characteristic parameters based on the SHAP model. Furthermore, the effect of the three supplementary cementitious materials on the flexural strength of concrete when they are combined with cement is investigated in turn for each of the three supplementary cementitious materials. The detailed technical route process is shown in Fig. 5.

## Results

### Parameter optimization

**Figure 6:**
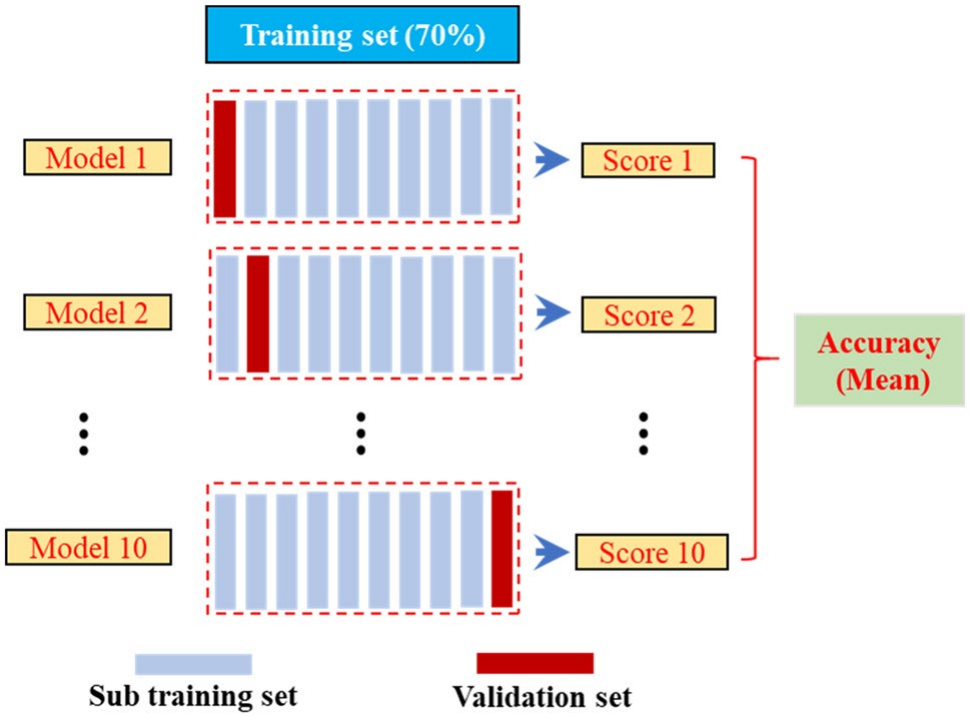
Ten-fold cross-validation schematic.

Parameter optimization is an essential step in machine learning and can be a good way to improve the prediction accuracy of machine learning. Parameter processing using PYTHON. The processing is carried out by grid search and ten-fold cross-validation. Grid search is performed through the original parameters in the model to seek the optimization direction. The optimal result is obtained after extensive processing. Ten-fold cross-validation was used in analyzing the calculation and its calculation schematic is shown in Fig. 6. There are many reports about the method, which will not be detailed here.

When performing parameter optimization, different models will have different optimization objectives. Among them, in the three machine learning models used in this study, the objects of their main optimization parameters are shown in Table 3. As can be seen from the table, two identical optimization objectives exist for the two models and two different optimization objectives also exist. The final optimization results obtained by the parameter optimization method described earlier are shown in the table.

**Table 3:** Optimization results of numerical indicators.

Group	Parameters	Values
RF	max depth	12
	n estimators	100
	min split	2
	min leaf	1
XGB	max depth	7
	n estimators	80
	gamma	0.65
	eta	0.31

### Predicted results

The prediction results of the three machine learnings used in this study in predicting the flexural strength of concrete are presented in Table 4. The LR model has the smallest value of R^2^. Furthermore, the LR model had the smallest PCC value and the rest of the metrics were the largest. This indicates that the LR model has an overfitting problem and has the worst prediction accuracy.

**Table 4:** Comparative results of model prediction effects.

Marks	LR model		RF model		XGB model	
	Train	Test	Train	Test	Train	Test
*R*^2^	0.57	0.47	0.96	0.87	0.98	0.93
*MAE*	1.34	1.37	0.37	0.72	0.32	0.55
*MSE*	3.13	3.38	0.22	0.87	0.16	0.52
*RMSE*	1.77	1.84	0.47	0.93	0.40	0.72
*PCC*	0.73	0.73	0.94	0.94	0.97	0.97

The two integrated learning models gave better predictions. In terms of R^2^ values, both the two models are larger and more similar. The variation in R^2^ between the two datasets was small, indicating that the models are not overfitted in the fitting process. In addition, for the XGB model, the FR model has larger values for all metrics except for the PCC value. In response to the above mathematical and statistical results, it can be concluded that the XGB model is most effective in predicting the flexural strength of concrete containing supplementary cementitious materials.

**Figure 7:**
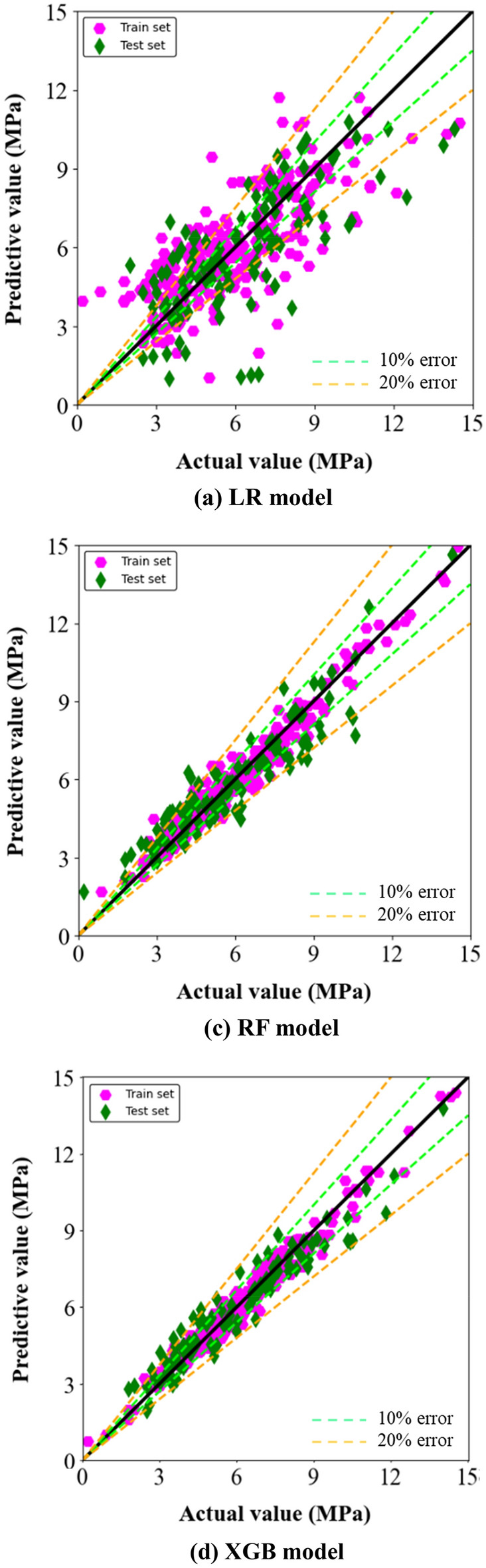
Machine learning prediction results for flexural strength of concrete.

In order to more intuitively characterize the effectiveness of the three machine learning models in predicting the flexural strength of concrete, the prediction results of the training and test sets are pooled in Fig. 7 in this study in the form of images. As can be seen from Fig. 7a, when using the LR model for prediction, the test set shows a characteristic of scattered distribution of data points around the equivalence points. Figure 7b shows the prediction results of the RF model, and it can be seen that the data points in the RF model are more concentrated than those in the LR model, with most of the data points on both sides of the equivalence point, and only a small number of data points are distributed in the error interval of more than 20% prediction results. It is clear from Fig. 7 that the XGB model has excellent results in predicting the flexural strength of concrete with supplementary cementitious materials.

### SHAP analysis

#### Computational thinking

The analytical model used in analyzing the effect of various parameters on flexural strength is SHAP^63^. The model has its origin in game theory and the effects of all the parameters are considered together in the analysis. In a particular prediction data, the model yields a prediction that can be labeled as SHAP value. The principle of this value is shown in Eq. (13):

13 $$f\left( {x_{i} } \right) = u_{0} + g\left( {x_{i1} } \right) + g\left( {x_{i2} } \right) + \ldots + g\left( {x_{ij} } \right)$$

*f*(*x*_*i*_) is the prediction result i; *u*_0_ is the model baseline; *x*_*ij*_ denotes the *j* parameter for sample *i*; *g*(*x*_*ij*_) is the devote in *f*(*x*_*i*_). *f*(*x*_*i*_) has the uniqueness^17^. In PYTHON, the calculation is analyzed by SHAP. Based on the data in the database, the feature values are obtained using the XGB model.

The SHAP value can characterize both the parameter with the highest degree of influence when analyzing the influence of a parameter and the influence process within the parameter range. In this case, the impact is positively correlated for SHAPM values greater than 0 and negatively correlated for values less than 0.

#### Analysis of impact elements

Based on the previous comparison of the computational results of the three machine learning models, it was demonstrated that the XGB model performed optimally in predicting the flexural strength of concrete. Therefore, in this study, the SHAP model was used to analyze the effect of various characteristic factors on the flexural strength of concrete based on the XGB model.

**Figure 8:**
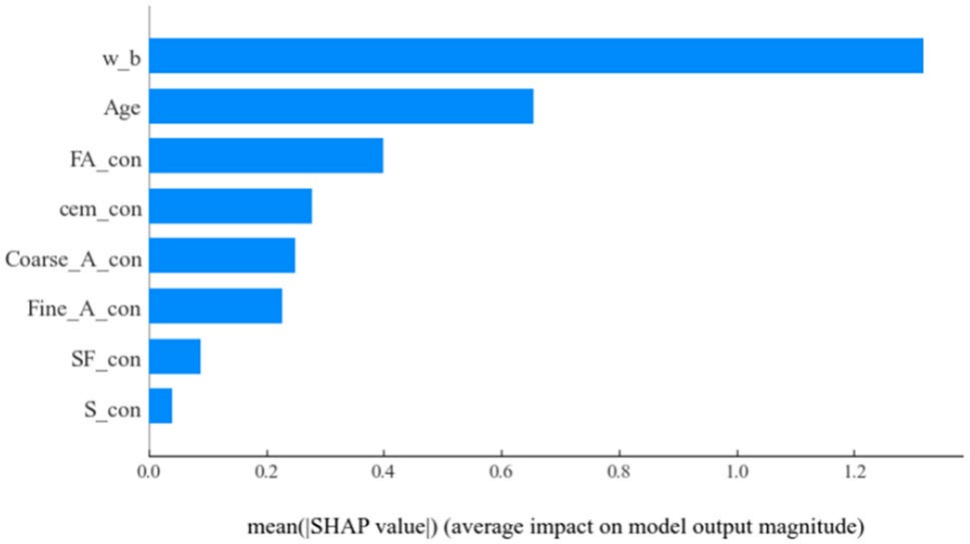
Average SHAP value of each characteristic parameter for concrete flexural strength prediction.

The gradient of the strength influenced by the eight input parameters affecting the flexural strength of concrete was first determined and characterized using SHAP mean values, and the results are shown in Fig. 8. It can be seen that for the flexural strength of concrete containing supplementary cementitious materials, the level of impact of the eight characteristic factors, in order from strong to weak, is: w_b, Age, FA_con, cem_con, Coarse_A_con, Fine_A_con, SF_con, S_con.

**Figure 9:**
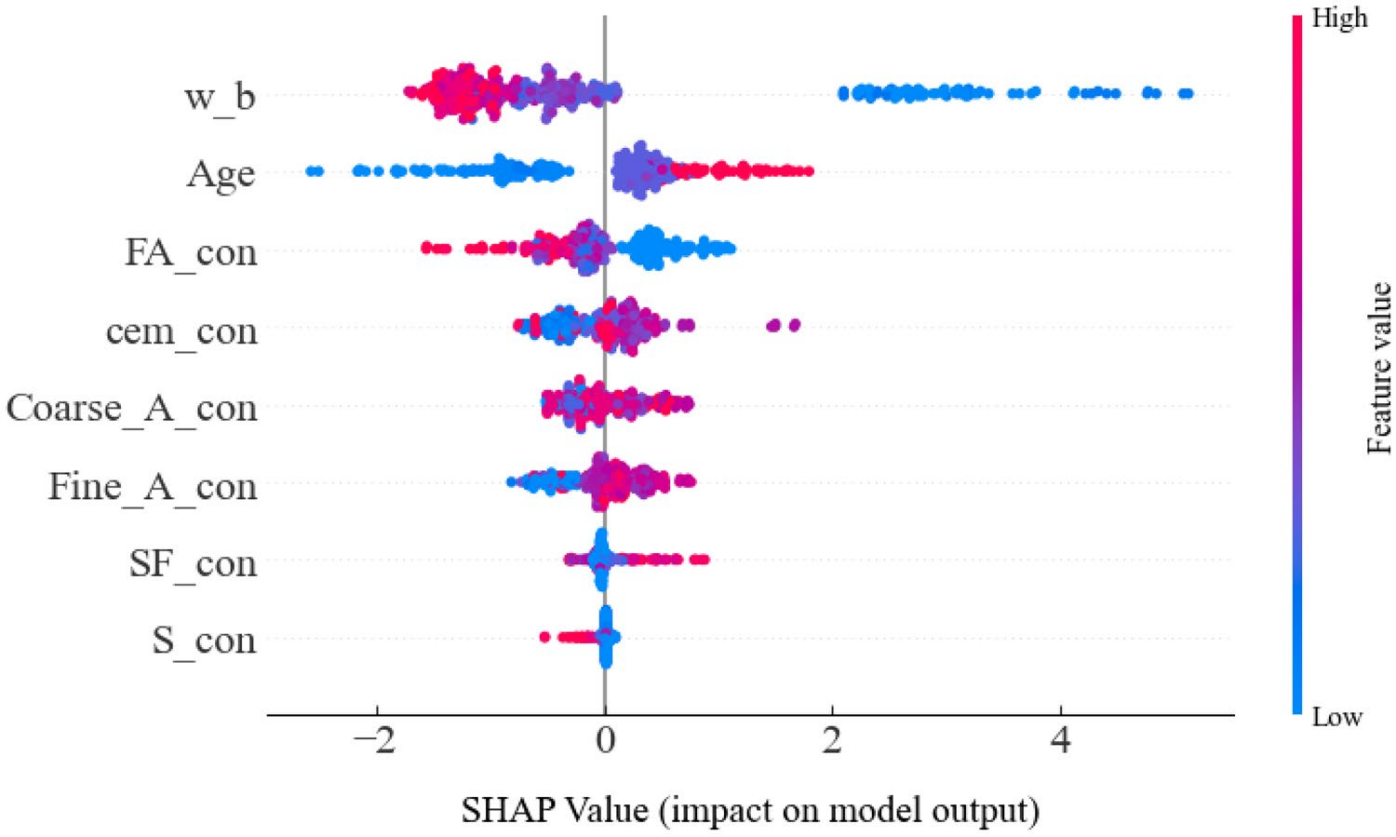
Analysis of the importance of various factors in the prediction of flexural strength of concrete using SHAP model characterization.

To further analyze the effects of various input parameters on the flexural strength of concrete and to provide insight into the trend of their effects on flexural strength from the numerical changes of the input parameters, the SHAP values of the input parameters shown in Fig. 9 were used for global interpretation in this study. The influence of each characteristic parameter on the flexural strength decreases in order. In the SHAP value, the relationship between the characteristic parameters and flexural strength can be assumed that there are two forms of correlation, one positive, i.e., the color red. One is a negative correlation, i.e., blue.

Combining Figs. 8 and 9, it can be found that w_b has the strongest relationship with the flexural strength of concrete containing supplementary cementitious materials. Moreover, as the SHAP value of w_b increases, w_b shows a negative trend of correlation with flexural strength. For Age and SF_con, the larger the SHAP value of both, the color of the data points gradually appears blue, which indicates that the above two input parameters show a strong positive correlation trend with the flexural strength. The remaining input parameters, compared to the above three input parameters, do not show a clear trend in the SHAP value global plot in relation to the flexural strength. Therefore, in order to fully interpret the relationship between the specific numerical variation of each input parameter and the flexural strength of concrete, this research will be analyzed and explained in detail.

#### Influence of various characteristic parameters

**Figure 10:**
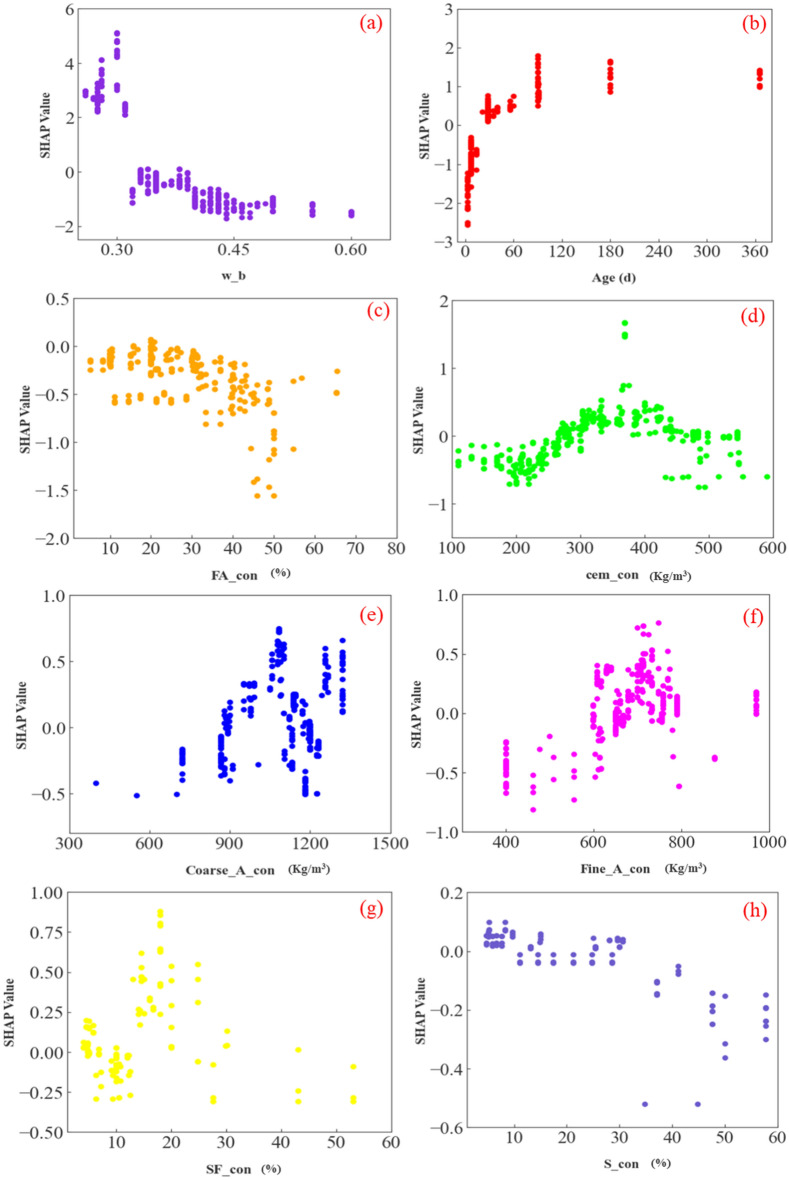
Influence of various parameters on flexural strength and the process of impacting them.

The relation of the effect of various parameters on the strength is illustrated in Fig. 10. The calculation results show that various characteristic parameters affect the SHAP value, but the degree of influence varies. First, there is the most significant effect on the flexural strength, w_b, as shown in Fig. 10a. From Fig. 10a, the SHAP value is gradually decreasing as w_b increases in the database of this study. This indicates that the strength is gradually decreasing with w_b. Moreover, the strength fluctuates more significantly when the w_b is between 0.3 and 0.35. In the images of the remaining characteristic parameters, the relationship between the data values of Age and SHAP value shows a completely opposite trend to w_b. That is, as Age increases, its SHAP value shows a clear upward trend. This indicates that strength increases with increasing age of concrete curing. As for the effect of cement admixture, it can be seen from Fig. 10d that when its content is between 200 and 600 kg/m^3^, the variation of SHAP values showed fluctuation with extreme values around 370 kg/m^3^. This indicates that the strength will have a peak value at a cement admixture of 370 kg/m^3^. However, since in this study, there is cement along with supplementary cementitious materials, the effect of cement needs to be analyzed together with the three supplementary cementitious materials. The SHAP values of the two aggregates show similar trends with the aggregate content. Analyzing Fig. 10e,f, it can be concluded that the increase in aggregate makes the flexural strength of concrete increase first and then decrease.

Then the effect of the three supplementary cementitious materials on the flexural strength of concrete is analyzed, and it can be found from the figure that FA_con and SF_con show a similar trend to w_b affecting the flexural strength of concrete on some intervals. This indicates that in terms of strength, the above two characteristic parameters have less flexural strength when they are present in concrete in larger amounts. However, in marked contrast to w_b, the fluctuation of the SHAP value of FA_con at less than 40% content is not obvious, and even up to 20% shows an increasing trend. This indicates that the flexural strength of concrete peaks at around 20% of FA_con content. For SF_con, its SHAP value appears to show fluctuations below 30%. and a smaller peak occurs around 10% and 30%, respectively. Subsequently, when the content of S_con exceeds 30%, the value decline. Therefore, combining the numerical changes of the SHAP value of S_con in several content zones, it can be concluded that the effect of S_con on the flexural strength of concrete may have peaks at 10% and 30% when the content is small. However, when the content of S_con exceeds 30%, the flexural strength decreases gradually. As for the characteristic parameter SF_con, when its content is below 18%, the value presents a fluctuating growth tendency. When the value of SF_con exceeds 18%, the trend is downward. The above shows that the maximum value of flexural strength of concrete may occur when the amount of silica fume in the cementitious material is 20%.

**Figure 11:**
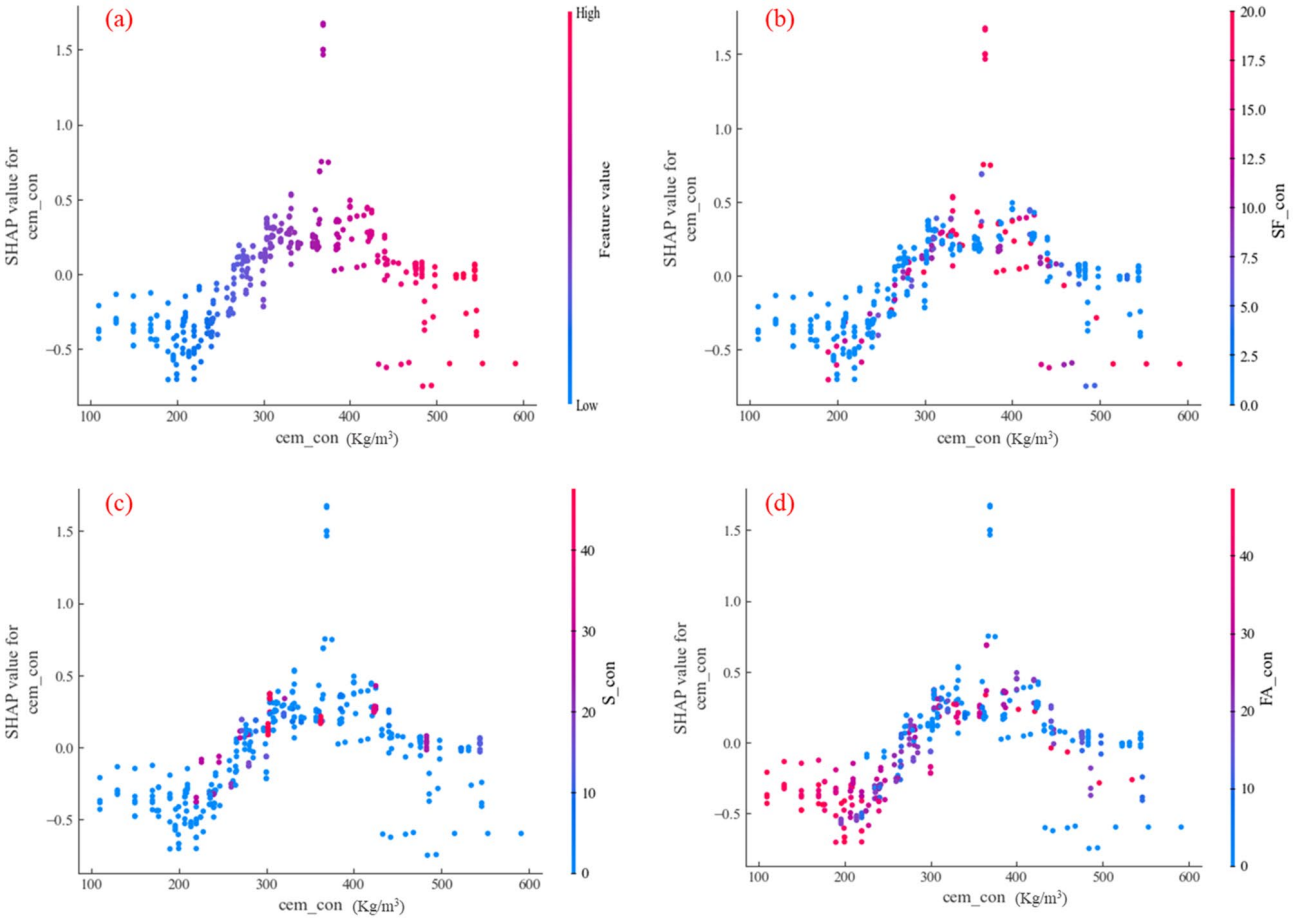
Interaction effects of supplementary cementitious materials.

In order to analyze in depth the effect of composite cementitious materials on the flexural strength of concrete when three supplementary cementitious materials act together with cement, and to clarify the interval of different supplementary cementitious materials acting on cement content. In this study, the SHAP values of the three supplementary cementitious materials were analyzed in combination with the SHAP values of the cement, the interactions were analyzed as shown in Fig. 11. Figure 11b presents the interaction effect of fly ash and cement for flexural strength. The red data points are more extensive and the data points at the maximum value of SHAP value of Cem_con also show red color. This indicates that the FA and cement are closely related, and the effect of FA on the flexural strength of concrete is greater at the optimum cement admixture. The relationship of the slag with the cement is shown in Fig. 11c. The red data points are mainly concentrated between the cement content of 200 kg/m^3^ and 400 kg/m^3^, indicating that the slag and cement interactive intervals are less than FA, and there is a certain effect on the flexural strength of concrete in this interval. In Fig. 11d, the distribution interval of red data points is more extensive, but mainly concentrated in the interval of cement less than 300 kg/m^3^. This indicates that the interaction interval between silica fume and cement is mainly concentrated in the interval when the cement content is small, and silica fume will have an important effect on the flexural strength of concrete in this interval.

## Conclusions

For the flexural strength of concrete containing supplementary cementitious materials, prediction studies were carried out using three machine learning models. The parameters were analyzed based on SHAP model, with the following main conclusions:

- The LR model is the poorest predictor of flexural strength for concrete with supplementary cementitious materials. The XGB model has a higher prediction accuracy than the RF model. The XGB model is recommended for predicting the flexural strength of concrete with cementitious admixtures.
- Among the characteristic parameters that affect the flexural strength of concrete with supplementary cementitious materials, the highest degree of correlation with the predicted results is the water-binder ratio, followed by the curing age. The weakest correlation with the predicted results was the admixture of silica fume and slag.
- Water-cement ratio and age of curing have the greatest influence on flexural strength. The water-cement ratio is negatively correlated with flexural strength, while the age of maintenance is positively correlated. The relationship between aggregate content and flexural strength showed a positive correlation followed by a negative correlation. The relationship between flexural strength and cement content was similar to that of aggregate content. The effect of the three supplementary cementitious materials on the flexural strength of concrete can be summarized as the correlation with flexural strength fluctuates at small admixtures (FA_con < 40%, SF_con, and S_con < 30%) and there is a positive correlation with the optimum admixture. However, the correlation becomes significantly negative at dosing levels greater than the above.
- In predicting the flexural strength of concrete, different supplementary cementitious materials interact with cement in different admixture intervals. Fly ash can act in the whole blending interval of cement. Slag has an important role between cement h content of 200 kg/m^3^ and 400 kg/m^3^. Silica fume has an important role in low cement content.

## Data Availability

Some or all data, models, or code that support the findings of this study are available from the corresponding author upon reasonable request.
